# Maternal Perinatal Outcomes Related to Advanced Maternal Age in Preeclampsia Pregnant Women 

**Published:** 2019-12

**Authors:** Bestari Dianing Tyas, Pudji Lestari, Muhammad Ilham Aldika Akbar

**Affiliations:** 1 Midwifery Student, Faculty of Medicine Universitas Airlangga, Surabaya, Indonesia; 2 Department of Preventive Medicine and Public Health, Faculty of Medicine Universitas Airlangga, Surabaya, Indonesia; 3 Department of Obstetrics and Gynecology, Faculty of Medicine Universitas Airlangga, Surabaya, Indonesia; 4 Department of Obstetrics and Gynecology, Universitas Airlangga Hospital, Surabaya, Indonesia

**Keywords:** Advanced Maternal Age, Preeclampsia, Maternal Outcomes, Perinatal Outcomes

## Abstract

**Objective:** This study aims to analyze the effect of advanced maternal age (>35 years old) in maternal and perinatal outcomes of preeclampsia women.

**Materials and methods:** This is a retrospective cross-sectional study involved all women who were diagnosed with preeclampsia at Universitas Airlangga Hospital (Surabaya, Indonesia) between January 2016 until May 2017. The participant was divided into two groups based on maternal ages: the first group was women older than 35 years old (advanced maternal age - AMA), and the other group was 20-34 years old (reproductive age - RA). The primary outcomes of this study were the maternal and perinatal outcome.

**Results:** There were a total of 43 AMA preeclampsia women and 105 RA preeclampsia women. The AMA preeclampsia group had a higher proportion of poor maternal outcome (the occurence of any complication: pulmonary edema, HELLP syndrome, visual impairment, post partum hemorrhage, and eclampsia) compared to RA preeclampsia group (60,5% vs 33,3%, p = 0,002; OR 3,059, CI 1,469-6,371). There was no significant difference in the other maternal complications such as HELLP syndrome, pulmonary oedema, and eclampsia. The only difference was the occurrence of postpartum haemorrhage which was higher in the AMA group (16,3% vs 4,8%, p = 0,02; OR 3,889, CI 1,161-13,031). The prevalence of cesarean delivery was more common in AMA group (53,3% vs 28,6%, p = 0,004; OR 2.825, CI 1.380-5.988). The AMA preeclampsia women also had poorer perinatal outcomes compared to the RA group (81,4% vs 59%, p = 0,009; OR 3.034 CI 1.283-7.177). AMA women had a higher risk of perinatal complication such as prematurity (OR 3.266 CI 1.269-8.406), IUGR (OR 4.474 CI 1.019-19.634), asphyxia (OR 4.263 CI 2.004-9.069), and infection (OR 2.138 CI 1.040-4.393).

**Conclusion:** Advanced maternal age increases the risk of poorer maternal and neonatal outcomes in preeclampsia patients. The addition of advanced maternal ages in preeclampsia should raise the awareness of the health provider, tighter monitoring, complete screening and early intervention if needed to minimize the risk of complications.

## Introduction

Preeclampsia is one of the major causes of maternal and perinatal deaths worldwide, especially in a low and middle-income country (1). The actual number and the proportion of preeclampsia in Indonesia increased significantly for the past 3 years (2). Preeclampsia is a specific syndrome of pregnancy due to the complex pathogenesis of placental insufficiency, angiogenic imbalance, oxidative stress, and endothelial damage (3). Preeclampsia is marked by increasing blood pressure after 20 weeks gestational age with one or more sign-symptoms of maternal complication or fetoplacental insufficiency (4). While the exact cause of preeclampsia is still continuously investigated, many risk factor and predisposing factors have been identified (5-7). 

One of the possible risk factors for preeclampsia is maternal ages. Women over 35 years old [Advanced Maternal Age (AMA)] have 4,5 fold risk of suffering preeclampsia compared to women aged 25-29 years (8). A similar study in China showed that women aged 35-39 years old and ≥ 40 years old had a higher risk of suffering preeclampsia 3,80 and 7,46 fold compared to normal reproductive age (9). Another study in Japan also found women aged > 45 years 1,86 times more likely to have preeclampsia and 2,03 times for severe preeclampsia (10). AMA was considered as an independent risk for poor outcomes in preeclampsia patients (11). Women with AMA more likely to have a preterm delivery before 37 weeks, before 34 weeks, low Apgar score, small for gestational ages, cesarean section, admission to NICU (11). The association between maternal ages and adverse pregnancy outcomes has been largely studied  (11-14). Several possible explanations included: the blood vessels ageing process, arterial stiffness, maternal hemodynamic adaptation impairment (8), lower ovum quality, obesity, unhealthy lifestyles, and comorbid disease (11). Although many studies about the effect of AMA on pregnancy outcomes have been conducted (15-17), evidence about outcomes in a specific group of patients with preeclampsia still less. A couple of studies has been performed to evaluate the relation between AMA and Gestational Hypertension (18, 19), but not with preeclampsia. And the other similar study only focused on the perinatal-neonatal outcome, without the maternal outcome. Therefore, the aim of this study was to compared pregnancy outcomes (maternal - perinatal) of AMA compared to normal RA (Reproductive Ages) preeclampsia patients.

## Materials and methods

**Figure 1:**
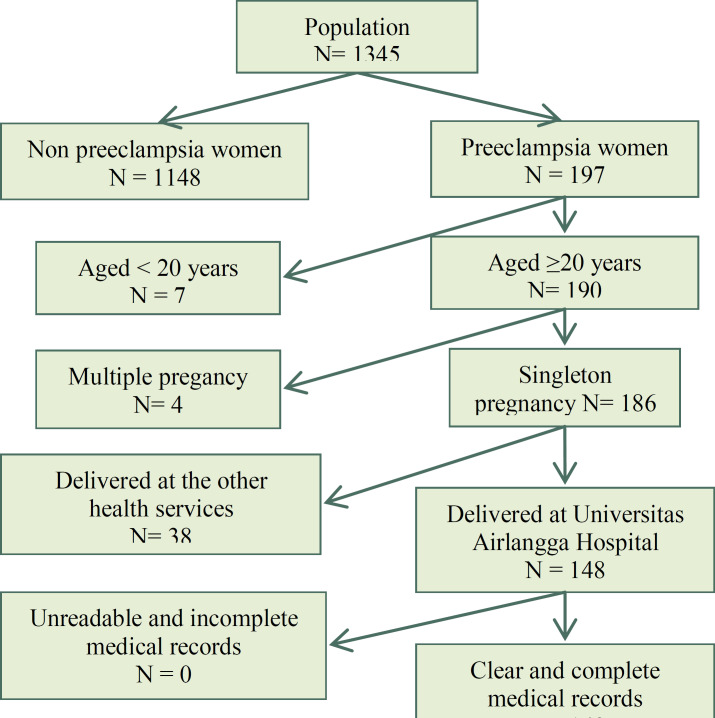
The sample recruitment process

This was a retrospective cross-sectional study conducted in Universitas Airlangga Hospital, Surabaya, Indonesia. The data used for this study was taken from medical records for total patients with preeclampsia which delivered in the hospital from January 2016 to May 2017 (total sampling). Inclusion criteria were preeclampsia women with a singleton pregnancy aged > 20 years, who delivered at Universitas Airlangga Hospital. While exclusion criteria were unreadable and incomplete medical records (Figure 1). The sample then divided into two groups based on maternal ages: AMA group (maternal age > 35 years old) and normal RA group (20-35 years old). Maternal age was confirmed based on the date of birth in the medical record. We compared the pregnancy outcomes of AMA preeclampsia group vs RA preeclampsia group. The ethical approval of this study has been given by Universitas Airlangga Hospital Ethics Committee (Number: 198/KEH/2018). The research process warranted anonymity and confidentiality by disregarded the respondent's name and used code on the data collection sheet. The report was submitted, stored and became Universitas Airlangga Hospital rights. 

Diagnosis of preeclampsia was taken from the medical record and confirmed based on the International Society for the Study of Hypertension in Pregnancy (ISSHP) criteria. Preeclampsia defined as persisting hypertension (blood pressure 140-159/90-109 mmHg) after 20 weeks gestation with urine protein/ creatinine ≥30mg/ mmol (0,3 mg/mg) or ≥ 300 mg/ 24 hours or at least 1g/L (2+) dipstick. Severe preeclampsia was defined as severe hypertension (blood pressure > 160/110 mmHg) occurs after 20 weeks gestation with one or more sign/symptoms of maternal organ dysfunction or uteroplacental insufficiency (IUGR) (4,20). Maternal organ dysfunction could be manifest as a renal failure/acute kidney injury (serum creatinine ≥ 90 umol/L; 1,02 mg/dL or at least 3+ dipstick), liver complications (increase level of SGPT/ SGOT 2 fold from normal limit, epigastric pain), neurological complications (eclampsia, decreased consciousness, blindness, cerebrovascular accident (CVA), hyper-reflection) and hematological complications (thrombocytopenia, Disseminated Intravascular Coagulation (DIC), hemolysis) (4). 

The primary outcomes of this study were maternal and perinatal outcomes. The maternal outcomes examined were poor maternal outcomes, maternal deaths, preeclampsia complications (HELLP syndrome, visual impairment, pulmonary oedema, and eclampsia), postpartum haemorrhage and mode of delivery. Poor maternal outcomes were diagnosed when the patients had minimal one maternal complication. Maternal deaths were diagnosed by death during the pregnancy period or within 42 days after delivery, due to all causes that are related or aggravated by pregnancy or treatment. HELLP syndrome was diagnosed as the special occurrence of new-onset hemolysis, thrombocytopenia, and elevated liver enzyme (Alanine Aminotransferase or Aspartate Transaminase levels) (20,21). Visual impairment was diagnosed from clinical symptoms and confirmed by an ophthalmologist. Acute pulmonary oedema was diagnosed from the clinical symptoms of severe respiratory distress and the finding of butterfly pattern on chest X-rays (22). Eclampsia was defined as preeclampsia complicated by a new onset of grand mal seizures before, during, or after labour (20). Postpartum haemorrhage was diagnosed as an excessive blood loss > 500 ml in vaginal delivery or > 1000 ml in cesarean delivery, including both primary or secondary type (23). 

The perinatal outcomes observed were poor perinatal outcomes, perinatal death, prematurity, SGA, asphyxia, low birth weight, Respiratory Distress Syndrome (RDS), infection, Necrotizing Enterocolitis (NEC), and Intraventricular Hemorrhage (IVH). The poor perinatal outcome was defined as one or more complication found after birth. Perinatal death was defined as an infant death on the first seven days of life. The diagnosis of perinatal death was taken directly from a maternal medical record and confirmed by the perinatal death record in the Pediatric Department of Universitas Airlangga Hospital. Prematurity was measured as preterm delivery < 37 weeks and preterm delivery < 34 weeks gestational age. IUGR was diagnosed using Ballard score and Lubchenco score for babies who were small for gestational age (24). Asphyxia was diagnosed by clinical examination show an APGAR score < 7 on the first 5 minutes. Low Birth Weight (LBW) was diagnosed if the baby birth weight less than 2500 grams. RDS determined by chest X-ray findings of hypoexpansion, ground-glass opacity, air-bronchogram sign, faded cardiac border or white lungs (25). Blood gas analysis showed hypoxic, hypercapnia, and oxygen partial pressure/amount of oxygen inhaled ≤ 26,7 kPa (25). Infection defined by clinical examination and blood culture (26). NEC diagnosed by clinical symptoms, biomarker test, and radiological features (27, 28). IVH was diagnosed by cranial ultrasonography at aged 3 days, 7 days and before discharged (29).

The data was analyzed using SPSS 25. The differences in proportion of between the two groups were analyzed with a Chi-Square test to evaluate the significance level (p < 0.05). The risk estimation was determined by calculation of the Odd Ratio (and 95% Confidence Interval (CI)) from the Chi-square test. Meanwhile, Contingency Coefficient (CC) with a significance of 5% was performed to determine the correlation among maternal age of preeclampsia women, maternal outcomes and perinatal outcomes. And multiple logistic regression with adjusted p value was performed to evaluate the effects of maternal and perinatal outcomes.

## Results

There was a total of 148 preeclampsia patient involved in this study, consisted of 43 women > 35 years old (AMA group) and 105 women 20-35 years old (RA group). The majority of participants was diagnosed as severe preeclampsia (AMA vs RA group: 88,4% vs 92,4%), and late-onset type (67,4% vs 73,3%). There was no significant difference between both groups in term of education background, employment, history of chronic hypertension, diabetes mellitus, diabetes gestational, body mass index, and haemoglobin level. The AMA group had a higher proportion of multipara compared to RA group (81,4% vs 65,6%, p = 0,0001). AMA group also had a higher proportion of women with a history of prior preeclampsia compared to RA group (25,6% vs 8,6%, p = 0,006). Small percentages of AMA and RA women had comorbidity of gestational diabetes (7% & 2,9%; p = 0,249), and history of previous diabetes (4,7% vs 3,8%; p = 0,814). The majority of patients from both groups had an abnormal BMI during initial visit, underweight or obesity (Table 1).

***Maternal Outcomes*** ***: ***There was no maternal death found in this study. The number of poor maternal outcomes was significantly higher in the AMA group compared to the RA group (60,5% vs 33,3%; p = 0.002). And AMA increased the risk of the poor maternal outcome in preeclampsia patients 3 fold higher (OR = 3,059; 95% CI = 1,469-6,371). Postpartum hemorrhage was found more often in AMA group compared to RA group (16,3% vs 4,8%; p = 0.02), with increased risk about 3,8 fold (OR = 3,889; 95% CI = 1,161-13,031).

**Table 1:** General Characteristics

**Characteristics**	**AMA** a ** Group** **n (%)** **(n** **=** **43)**	**RA** b ** Group** **n (%)** **(n** **=** **105)**	**P** ** value**
Classification of Preeclampsia			0.434
Severe preeclampsia	38 (88.4)	97 (92.4)	
Preeclampsia	5 (11.6)	8 (7.6)	
Onset of preeclampsia			0.470
Early onset (< 34 weeks)	14 (32.6)	28 (26.7)	
Late onset (> 34 weeks)	29 (67.4)	77 (73.3)	
Employment			0.288
Work	17 (39.5)	32 (30.5)	
Does not work	26 (60.5)	73 (69.5)	
Parity			< 0.001*
Primipara	4 (9.3)	36 (34.3)	
Multipara	35 (81.4)	69 (65.7)	
Grandemultipara	4 (9.3)	0 (0)	
Prior preeclampsia			0.006*
Yes	11 (25.6)	9 (8.6)	
No	32 (74.4)	96 (91.4)	
Prior chronic hypertension			0.076
Yes	14 (32.6)	20 (19)	
No	29 (67.4)	85 (81)	
Prior diabetes mellitus			0.814
Yes	2 (4.7)	4 (3.8)	
No	41 (95.3)	101 (96.2)	
Diabetes Gestasional			0.249
Yes	3 (7)	3 (2.9)	
No	40 (93)	102 (97.1)	
Body Mass Index (BMI)			0.056
≤ 19,9 kg/m^2^	2 (4.7)	12 (12.4)	
20-24,9 kg/m^2^	14 (32.6)	48 (45.7)	
25-29,9 kg/m^2^	22 (51.2)	30 (28.6)	
≥ 30 kg/m^2^	5 (11.6)	14 (9.5)	
Hemogloblin Levels			0.928
< 11 gr/dL	13 (30.2)	30 (28.6)	
11-12,9 gr/dL	27 (62.8)	69 (65.7)	
≥ 13 gr/dL	3 (7)	5 (5.7)	

a Advanced Maternal Age;

b Reproductive Ages;

* indicate significance level, p < 0.05

**Table 2:** Maternal Outcomes

**Maternal Outcomes**	**AMA** a ** group** **(n=43)** **n (%)**	**RA** b ** group** **(n=105)** **n (%)**	**P value**	**CC**	**OR** **(** **95% ** **CI)**	**Adjust** **ed P ** **Value**	**OR** **(95% CI)**
Poor Maternal outcome			0.002*	0.243	3.06 (1.47-6.37)	0.002*	0.75 (0.15-3.70)
Yes	26 (60.5)	35 (33.3)					
No	17 (39.5)	70 (66.7)					
HELLP syndrome			0.650	-	-	0.638	-
Yes	1 (2.3)	4 (3.8)					
No	42 (97.7)	101 (96.2)					
Visual impairment			0.589	-	-	0.597	-
Yes	3 (7)	5 (4.8)					
No	40 (93)	100 (95.2)					
Pulmonary edema			0.511	-	-	0.531	-
Yes	1 (2.3)	1 (1)					
No	42 (97.7)	104 (99)					
Postpartum hemorrhage			0.020*	0.188	3.89 (1.16-13.03)	0.027*	6.07 (1.10-33.41)
Yes	7 (16.3)	5 (4.8)					
No	36 (83.7)	100 (95.2)					
Eclampsia			0.869	-	-	0.871	-
Yes	1 (2.3)	2 (1.9)					
No	42 (97.7)	103 (98.1)					
Mode of delivery			0.004*	0.230	2.88 (1.38-5.99)	0.005*	4.15 (0.86- 20.04)
Cesarean delivery	23 (53.5)	30 (28.6)					
Vaginal delivery	20 (46.5)	75 (71.4)					

a Advanced Maternal Age;

b Reproductive Ages;

* indicate significance level < 0.05

Patients in the AMA group had a higher incidence of cesarean section compared to the RA group (53,5% vs 28,6%, p = 0.004). There were no significant differences in other maternal complications such as HELLP syndrome, visual impairment, pulmonary oedema and eclampsia. Based on the multiple logistic regression, the maternal outcomes which is correlated with AMA were only postpartum hemorrhage and mode of delivery (Table 2).

***Perinatal Outcomes*** **: **The incidence of poor perinatal outcome was significantly higher in AMA group (81,4% vs 59%, p = 0.009), and the risk was increased by AMA about 3 fold higher (OR = 3,034; 95% CI = 1,283-7,177). AMA group had significantly higher proportion of preterm birth < 37 weeks (25,6% vs 9,5%, p =0.011), IUGR (11,6% vs 2,9%, p = 0.032), Asphyxia (55,8% vs 22,9%, p = 0.0001), and perinatal infection (55,8% vs 37,1%, p = 0.037). There was no significant difference in the percentage of preterm birth < 34 weeks, low birth weight and respiratory distress syndrome between both group (p > 0.05). There was no incidence of perinatal death and other perinatal complications such as intraventricular hemorrhage and *necrotizing enterocolitis *in this study. Based on the multiple logistic regression, the perinatal outcomes which is correlated with AMA were preterm delivery < 37 weeks, IUGR, asphyxia, and infection (Table 3).

## Discussion

***Maternal Outcomes*** **: **This study confirmed that AMA is one of the important risk factors for poor maternal outcomes in preeclampsia cases. Preeclampsia women complicated by AMA had a higher risk of poor pregnancy outcomes, cesarean delivery and postpartum haemorrhage, although the risk of preeclampsia complication itself (HELLP syndrome, eclampsia, pulmonary oedema, and visual impairment) was not increased.

AMA group had a risk of poor maternal outcome 3 fold higher compared to the RA group. This result was in line with a large retrospective cohort study in china involving 2800 singleton pregnancy, finding increased adverse pregnancy outcomes related to increasing maternal ages (9). The possible explanation for these findings might be related to the higher proportion of overweight-obesity women in the AMA group (62,8% vs 38,1%). 

**Table 3:** Perinatal Outcomes

**Perinatal Outcomes**	**AMA** a ** group** **n (%)** **(n** **=** **43)**	**RA** b ** group** **n (%)** **(n** **=** **105)**	**P value**	**CC**	**OR** **(CI 95%)**	**Adjuste** **d P ** **Value**	**OR** **(95% CI)**
Poor Perinatal outcome			0.009*	0.20 9	3.03 (1.28-7.18)	0,007*	0,58 (0,15-2,25)
Yes	35 (81.4)	62 (59)					
No	8 (18.6)	43 (41)					
Preterm delivery (< 34 weeks)			0.521	-	-	0.406	-
Yes	0 (0)	1 (1)					
No	43 (100)	104 (99)					
Preterm delivery (<37 weeks)			0.011*	0.20 5	3.27 (1.27-8.41)	0.015*	2.84 (0.94-8.55)
Yes	11 (25.6)	10 (9.5)					
No	32 (74.4)	95 (90.5)					
IUGR			0.032*	0.17 3	4.47 (1.02-19.63)	0.043*	5.13 (0.97-27.06)
Yes	5 (11.6)	3 (2.9)					
No	38 (88,4)	102 (97.1)					
Asphyxia			< 0.001*	0.30 4	4.26 (2.00-9.07)	<0.001*	4.35 (1.68-11.24)
Yes	24 (55.8)	24 (22.9)					
No	19 (44.2)	81 (77.1)					
Low birth weight			0.899	-	-	0.898	-
Yes	7 (16.3)	18 (17.1)					
No	36 (83.7)	87 (82.9)					
RDS			0.589	-	-	0.597	-
Yes	3 (7)	5 (4.8)					
No	40 (93)	100 (95.2)					
Infection			0.037*	0.16 9	2.14 (1.04-4.39)	0.038*	2.09 (0.78-5.62)
Yes	24 (55.8)	39 (37.1)					
No	19 (44.2)	66 (62.9)					

a Advanced Maternal Age;

b Reproductive Ages;

* indicate significance level< 0.05

Obesity may contribute to poor pregnancy outcomes, associated with increased risk of miscarriage, diabetes gestational, hypertension in pregnancy, SGA, and cesarean section (15, 30). Although the detailed mechanisms for this relationship still need to be further investigated (30). Another possible supporting factor for the poorer pregnancy outcomes in the AMA group included preeclampsia type. Early onset preeclampsia proportion was higher in the AMA group compared to the RA group (32,6% vs 26,7%). A large cohort study has shown that early-onset preeclampsia increased the risk of maternal morbidity compared to late-onset preeclampsia, manifest as a cardiovascular, respiratory, central nervous system, hepatorenal, and other morbidities (31). The relatively higher number of chronic hypertension in the AMA group (32,6% vs 19%) could also contribute to the poorer maternal outcome. In another large cohort study of chronic hypertension in pregnancy, the occurrence of this comorbid was associated with maternal death and complications (32). 

 Our research shows that the risk of postpartum haemorrhage (PPH) was 3,9 fold higher in the AMA group compared to the RA group. The two large studies on AMA effect in pregnancy outcome did not mention about the PPH rate (11, 15), but Khalil et al study reveals no significant difference on blood transfusion rate between AMA dan RA group (15). Need for blood transfusion can be seen as an indirect consequence of severe PPH. We argued that the higher risk of PPH in AMA group could result from the higher incidence of anaemia (30,2% vs 28,6%), which is an important risk factor of PPH (30,2% vs 28,6%) (23). Another possible caused of this higher rate of PPH could be from the higher proportion of overweight and obese women (> 50%) and the higher parity in AMA group, which is also risk factors of PPH (23). All of the PPH in the AMA group happened in multipara pregnant women. This study also confirmed other large study finding that AMA increased the risk of cesarean delivery more than 2 fold. In the Finnish registry-based study and UK study, AMA increased the risk of cesarean delivery (OR: 2.02 vs 1.95), similar to our finding (11, 15), Mylonas et al. mentions different results to our study that AMA was not a direct indication for cesarean delivery, but it more related to the comorbidities which occurred in AMA pregnant women (33).

The other important outcomes such as maternal death and preeclampsia complications (HELLP syndrome, visual impairment, pulmonary oedema, and eclampsia) did not show any difference between both groups. This was an important finding since large Finish and UK studies did not evaluate these outcomes (11, 15). While advanced maternal age could be an independent risk factor for preeclampsia or hypertension in pregnancy (15), this study showed that it did not predispose to the development of severe complication in already sick preeclampsia women.

***Perinatal Ou*** ***tcomes: ***This study revealed that AMA increased the risk of poor perinatal outcomes, preterm birth < 37 weeks, IUGR, asphyxia, and perinatal infection in preeclampsia pregnant women. Poor perinatal outcomes were found 3 times higher in the AMA group compared to the RA group. This overall combined poor perinatal outcomes rate was not evaluated in the other study. The higher poor perinatal outcomes in the AMA group could be contributed by the higher number of early-onset preeclampsia and overweight-obesity patient. The early onset preeclampsia was associated with worse perinatal outcomes related to the severity of the disease, and the need to do early termination result in preterm delivery (34). Obesity also contributed to the higher risk of poor perinatal outcomes, included: infant mortality, low Apgar score, and asphyxia (30). The weaker adaptation of maternal cardiovascular function and impaired vascular function was found in advanced maternal age (35) and could be an important pathogenesis of poor perinatal outcomes. The impaired systemic and uterine arteries have a direct consequences of lower uteroplacental perfusion lead to higher rate of perinatal complications. 

The risk of preterm birth < 37 weeks was significantly increased 3,2 fold (95% CI: 1,269-8,406) in the AMA group. This was higher compared to the Finnish study which found an OR 1,39 (11). Preterm birth risk increased gradually correspond to the maternal age by any indication (spontaneous or iatrogenic)  (14, 36). Placental and myometrial vascular lesions, progesterone deficiency, increased miscarriage rates, smoking and overweight could contribute to the increased risk of preterm birth in the AMA group (36). The higher comorbidities found in the AMA group could also contribute to the higher number of preterm delivery. Shan D. et al study showed an interesting opposite result with this study, that AMA was a protective factor for preterm delivery in total patients involving preeclampsia cases (9). They argued that AMA pregnant women had a better quality of the embryos that can survive and avoid adverse outcomes in this high-risk pregnancy (9). 

The risk of IUGR was also higher in the AMA group compared to RA over 4 times. Estimate risk of IUGR in this study was significantly higher compared to similar studies in the United States, Finnish and Israel (OR 1,26 vs 1,42 vs 1,51) (11, 37, 38). In addition, studies in Taiwan revealed different results that the risk of SGA was declined with increasing maternal age  (14). The correlation between AMA and IUGR is still uncertain, but Odibo et al have confirmed that AMA was an independent risk factor for IUGR and both showed positive dose-response association (39). The comorbid factors commonly found in AMA could be a potential correlating factor between AMA and IUGR. While maternal underweight could be a risk factor for IUGR, in this study we did not found any different on the maternal underweight rate between both groups. The impaired uterovascular function correlated with increased maternal age could predispose to the development of IUGR (35). These findings suggest that the screening and early intervention of IUGR and preterm delivery in AMA pregnant women during antenatal care should be routinely performed. 

The incidence of asphyxia was 2 fold higher in the AMA group compared to the RA group, with over 4 fold increased risk. This finding is similar to the Finnish study, which stated that AMA was an important risk for asphyxia (11). Asphyxia was assessed from the Apgar score that could be influenced by many factors: fetal distress, intrauterine asphyxia, airway obstruction, CNS depression, prematurity, and use of sedative drugs (40). The possibility of uteroplacental insufficiency related to impaired vascular function could be an explanation (35). Another interesting finding in this study included the risk of infection which was 2 fold higher in the AMA group compared to the RA group. The possible cause of this might be related to the higher incidence of preterm delivery and asphyxia in the AMA group, which is a major risk of infection. Prematurity (especially in LBW) was associated with immaturity of innate immune system function predisposed to infection (41). There was no significant difference in the incidence of preterm delivery < 34 weeks, low birth weight, and RDS in this study. And there was no incidence of perinatal death, NEC, and IVH.

This study revealed that advanced maternal age increased the risk of poor maternal and perinatal outcomes in preeclampsia women. The strength of this study was the pregnancy outcomes in both maternal and perinatal side was evaluated in a specific group of preeclampsia pregnant women. While other study only analise the effect of AMA in general pregnant women. The result of the study suggest that antenatal care on this group of preeclampsia needs to be more intensive to prevent the occurrence of maternal and fetal complications. Screening for the risk of IUGR and preterm delivery should be performed routinely and early intervention if needed. Management of newborn in an advanced maternal age needs special attention to avoid the risk of perinatal infection, asphyxia, and perinatal death.

## Conclusion

Advanced maternal age (> 35 years) is an independent risk factor for poor maternal and perinatal outcomes in preeclampsia pregnant women. These studies emphasize that perinatal complications were more profound compared to maternal. This information could be important for health care providers in conducting early screening, intervention, or referral to the tertiary centre to prevent complications in the AMA group. Complete screening during antenatal care especially for the risk of IUGR and preterm delivery need to be performed regularly. In a low resource setting, early referral to the higher tertiary center (with a complete NICU facility) needs to be considered in preeclampsia patient with advanced maternal age, to anticipate the perinatal complications.
